# The Clinical Efficacy of Chemotherapy Combined with Traditional Chinese Medicine in the Treatment of Cervical Cancer and Its Influence on Cellular Immunity, Serum CEA, and TNF-*α*

**DOI:** 10.1155/2022/7728739

**Published:** 2022-01-07

**Authors:** Qiannan Zhao, Juanjuan Liu, Sai Wang, Xiuqin Wang, Xiufang Jiang

**Affiliations:** ^1^Department of Clinical Laboratory, Yantaishan Hospital, Yantai 26400, China; ^2^Department of Obstetrics, The Third People's Hospital of Qingdao, Qingdao 266041, China; ^3^ICU, Affiliated Qingdao Central Hospital, Qingdao University, Qingdao 266000, China; ^4^Department of Gynaecology, People's Hospital of Huakang, Rizhao 276800, China; ^5^Department of Obstetrics, Zhangqiu District People's Hospital, Jinan 250200, China

## Abstract

**Background:**

This study aims to investigate the clinical efficacy of chemotherapy combined with traditional Chinese medicine in patients with cervical cancer and its effect on cellular immunoglobulin, serum sugar chain antigen 125 (CA125), carcinoembryonic antigen (CEA), and tumor necrosis factor-*α* (TNF-*α*).

**Methods:**

Conventional chemotherapy was performed in control and observation groups. Meantime, the observation group received traditional Chinese medicine. Finally, the clinical efficacy, immunoglobulin, serum tumor markers, and serum TNF-*α* of the two groups were compared.

**Results:**

Compared with the control group, total effective rate in the observation group was increased. After treatment, serum CD8+, TNF-*α*, CA125, and CEA levels were reduced in the two groups, and the observation group was higher. In the two groups, CD3+ and CD4+ levels were enhanced after treatment, and the observation group was also higher. Compared with the control group, the immunoglobulin IgG, IgA, and IgM levels increased in the observation group. The incidence of adverse reactions in the observation group was reduced compared to the control group.

**Conclusion:**

Chemotherapy combined with traditional Chinese can help improve the clinical efficacy and immunity in patients with cervical cancer. Moreover, the safety and feasibility of the treatment method are relatively high.

## 1. Introduction

Cervical cancer (CC) is a common type of gynecological malignant tumor, and its development is a slow and continuous process from quantitative change to qualitative change [1–3]. There can be no symptoms in the early stage of CC. As the disease progresses, patients may experience symptoms such as contact bleeding and abnormal vaginal discharge [4, 5]. CC occupies the second place among the deaths of female malignant tumors in China, and its prevalence ranks first among female genital malignant tumors. According to the statistics of global cancer data in 2018, there are more than 560,000 newly diagnosed cases worldwide each year, and the death toll exceeds 310,000 [6]. The causes and pathogenic mechanisms of cervical cancer are very complicated. However, with the development technology, the cause of cervical cancer has gradually become clear. It has been confirmed that persistent HPV virus is the main cause of cervical cancer [7–11].

Recently, the diagnosis rate of CC is rising. However, the patient is already in the advanced stage at the time of diagnosis because of the stealthiest of CC incidence. Surgical treatment alone is less effective and has a lower survival rate for advanced patients. At present, the main treatment methods for CC include surgery, neoadjuvant chemotherapy [12–14], concurrent chemotherapy, radiotherapy, and short-term radiotherapy. Radiotherapy combined with systemic chemotherapy is often used for patients with advanced, recurrence, and metastasis CC. Chemotherapy includes adjuvant chemotherapy, consolidation chemotherapy, induction chemotherapy, intensive chemotherapy, maintenance chemotherapy, and neoadjuvant chemotherapy. Clinically, it has been found that patients with CC have different treatment effects due to the differences in the sensitivity of chemotherapy. Although increased dose of chemotherapy can improve the efficacy to a certain extent, it increases the occurrence of adverse reactions and side effects in patients. The increase of adverse reactions will affect the effect of chemotherapy, increase the suffering of patients, and affect the life quality of patients.

Traditional Chinese medicine [15–17] can not only enhance the antitumor effect of chemotherapy drugs but also improve the immunity of cancer patients. The antitumor effect of Chinese medicine mainly include inhibition of tumor cell proliferation, promotion of cell apoptosis, and differentiation and improvement of body immunity [18–21]. Combined treatment can obviously prevent recurrence and metastasis, reduce the damage of radiotherapy and chemotherapy to the digestive tract and hematopoietic system, and strengthen the effect of radiotherapy and chemotherapy [22–26]. Therefore, we explored the clinical efficacy of chemotherapy combined with traditional Chinese medicine in patients with CC and its influence on cellular immunity, serum TNF-*α*, CA125, and CEA.

## 2. Materials and Methods

### 2.1. Clinical Patients

78 patients with CC in Zhangqiu District People's Hospital from January 2018 to January 2019 participated in this research. This study was approved by the Ethics Committee of Zhangqiu District People's Hospital (2018-H15) and was performed based on the Declaration of Helsinki. In general information, no significant difference was found between the two groups (*P* > 0.05, Table 1).

#### 2.1.1. Inclusion Criteria

1. Western medicine diagnosis: symptoms and pathological examination results are in line with the “International Federation of Obstetrics and Gynecology Guidelines for the Diagnosis and Treatment of Cervical Cancer in 2015” [27]
2. The CC was diagnosed by cytological examination and pathological biopsy
3. Traditional Chinese medicine (TCM) diagnosis: all patients meet the “Guiding Principles for Clinical Research of New Chinese Medicines” [28]
4. Chemotherapy for the first time
5. Expected survival time is ≥6 months
6. Patients have complete clinical data
7. Patients provided written informed consent

#### 2.1.2. Exclusion Criteria

1. Patients with other malignancies
2. Liver, kidney, or other vital organ dysfunctions
3. Mental or blood system diseases
4. Patients are allergic to the drugs in our research
5. Treatment program was intolerant or refused to cooperate with this study

### 2.2. Treatment Methods

The control group received conventional chemotherapy. Before chemotherapy, the patient was treated with water chemotherapy for 3 days (liquid volume ≥3500 ml). Then, cisplatin (DDP) was continuously administered for the first 5 days of chemotherapy (40 mg/m^2^). Bleomycin (BLM) was given in the first 3 days (15 mg/m^2^). The observation group received simultaneous chemotherapy and traditional Chinese medicine. It has been taken since the first day of chemotherapy. Chinese medicine mainly focuses on clearing away heat and detoxification, invigorating the spleen and kidney, and nourishing blood. The prescriptions are as follows: *Codonopsis* 15 g, *Astragalus* 30 g, *Atractylodes* 15 g, *Poria* 15 g, *Angelica* 15 g, *Rehmannia* 15 g, *Lycium barbarum* 15 g, Psoralen 10 g, Sichuan *Dipsacus* 10 g, *Oldenlandia diffusa* 30 g, *Smilax glabra* 15 g, August stick 20 g, and Sunburn Licorice 6 g. Patients with nausea and vomiting were added *Evodia* 9 g. Patients with bloating and loss of appetite were added 15 g of fried malt, 15 g of fried grain sprouts, 9 g of tangerine peel, 10 g of roasted chicken inner gold, and 6 g of *Citrus aurantium*. Patients with insomnia were added 15 g Shouwu vine, 10 g Baiziren, and 3 g *Polygala*. Patients with cold stomach were add 3 g cloves and 6 g dried ginger. Patients with obvious fatigue were added 20 g of *Astragalus*.

#### 2.2.1. Observation Indicators

The clinical efficacy of patients includes complete remission (CR, complete regression of the lesion for more than one month), partial remission (PR, reduction of the lesion by >50% for more than one month), stable disease (SD), and disease progression (PD) [29].

(1) $$\begin{array}{c} \text{Total effective rate}=\frac{\left(\text{CR}+\text{PR}\right)}{\text{total}}\times 100\%. \end{array}$$

#### 2.2.2. Enzyme-Linked Immunosorbent Assay (ELISA)

5 ml of fasting venous blood was taken and centrifuged at 3000 r/min for 10 min. The supernatant was aspirated into the EP tube with a pipette. The i4000SR automatic immunoassay analyzer (Abbott, USA) was used to measure CA125, CEA, and TNF-*α* levels with special kits.

### 2.3. Cellular Immunoglobulin

The fluorescence molecular labeling method and flow cytometer were applied to detect immunoglobulin IgG, IgA, and IgM levels in peripheral blood.

### 2.4. T Lymphocyte Subgroup Detection

The flow cytometer (CyFlow® Cube8, Partec, Germany) and fluorescent molecular labeling method were used to detect the peripheral blood T lymphocyte subsets CD3+, CD4+, and CD8+. We calculated the CD4/CD8 ratio.

### 2.5. Statistical Analysis

All experiments were repeated 3 times. SPSS 22.0 software was used to analyze experimental data. Data are expressed as mean ± SD. The count data are expressed in *n* (%). The *χ*^2^ test was used for comparison. The difference is defined at *P* < 0.05.

## 3. Results

### 3.1. Comparison of Clinical Efficacy

The clinical symptoms of the two groups were relieved after treatment. A short-term effect was observed 1 month after treatment. The observation group included 11 CR patients (28.2%), 24 PR patients (61.5%), 3 SD patients (7.7%), and 1 PD patient (2.6%). The clinical efficacy rate was 89.7% (35/39). In the control group, there were 9 CR (23.1%), 25 PR (64.1%), 4SD (10.2%), and 1 PD (2.6%). The clinical efficacy rate was 87.2% (34/39). No significant difference was found in short-term clinical efficacy between the two groups (*P* > 0.05, Table 2).

### 3.2. Comparison of Serum Tumor Marker before and after Treatment

**Figure 1:**
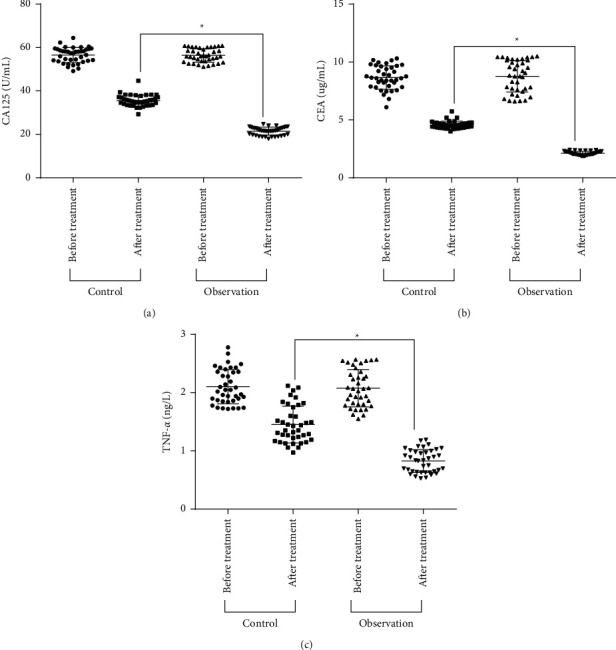
Comparison of serum tumor marker levels before and after treatment in the two groups. (a–c) CA125, CEA, and TNF-*α* levels were compared between two groups of patients (*n* = 39). ^*∗*^*P* < 0.05.

After treatment, the serum TNF-*α*, CEA, and CA125 levels in the two groups decreased. The levels of CA125, CEA, and TNF-*α* in the observation group were reduced compared to the control group (*P* < 0.05, Figure 1). The possible reason is that traditional Chinese medicine may promote the production of immunosuppressive cytokines in CC, thereby suppressing the local cervical immune response. The results suggest that traditional Chinese medicine has anti-inflammatory and analgesic effects by inhibiting the production of CA125, CEA, and TNF-*α*.

### 3.3. Comparison of T Lymphocyte Subsets

After treatment, CD4+ and CD4+/CD8+ in the cervical tissues of the two groups were increased. Meanwhile, the CD8+ level in the two groups was reduced (*P* < 0.05, Table 3). Compared to the control group, the increased level of CD4+ and CD4+/CD8+ and decreased level of CD8+ were detected in the observation group (*P* < 0.05, Table 3).

### 3.4. Comparison of Serum Immunoglobulin Levels before and after Treatment

After treatment, IgG, IgA, and IgM levels in the observation group were significantly higher than those in the control group (*P* < 0.05, Table 4). The increase in the patient's immune globulin level can effectively reflect the increase in the patient's immune level, further confirming the effectiveness of the drug.

### 3.5. Comparison of Adverse Reaction

**Figure 2:**
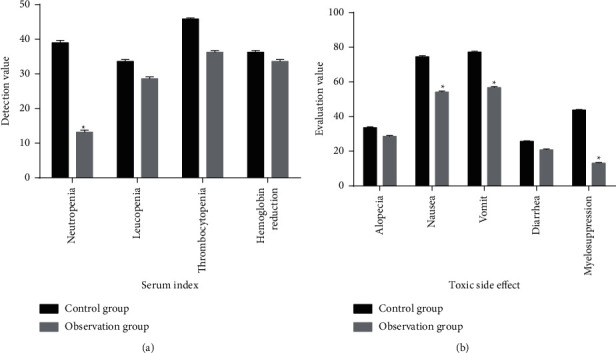
Comparison of adverse reactions between the two groups. (a) Comparison of serum toxicity and side effects between the two groups (%, *n* = 39). (b) The occurrence of clinical toxic and side effects compared between the two groups of patients (%, *n* = 39). ^*∗*^*P* < 0.05.

Before and after treatment, the patient's serological indicators were detected as indicators for monitoring serological side effects. Neutropenia was significantly reduced in the observation group compared with the control group (*P* < 0.05, Figure 2(a)). Thrombocytopenia, hemoglobin counts, and leucopenia decreased in both groups. However, there was no statistically difference between the two groups (*P* > 0.05, Figure 2(a)). The incidence of nausea, vomit, and myelosuppression in the observation group was lower than that in the control group (*P* < 0.05, Figure 2(b)). The results indicate that this treatment has high safety and is well tolerated.

### 3.6. Comparison of Life Quality Scores

**Figure 3:**
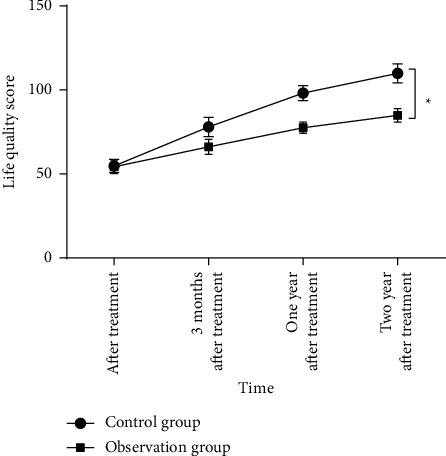
Comparison of life quality scores between the two groups (*n* = 39). ^*∗*^*P* < 0.05.

The life quality score in the observation group after treatment was higher than before treatment (*P* < 0.05, Figure 3). Similarly, high life quality score of patients was also found in in the control group after treatment (*P* < 0.05, Figure 3). Compared to the control group, the observation group had a higher life quality score after treatment (*P* < 0.05, Figure 3). The life quality score survey shows that chemotherapy and traditional Chinese medicine can improve the life quality of patients.

## 4. Discussion

World Society of Epidemiology and Statistics has proposed that CC is a malignant tumor that seriously affects women [1] in worldwide. Its risk severity is second only to breast cancer, and its growth momentum is higher than breast cancer [2]. CC has grown rapidly in worldwide in recent years, and China occupies a huge proportion. Due to the decline in environmental levels and diet quality, the high incidence of cancer is becoming one of the major problems that increasingly plague mankind [3]. Meanwhile, the incidence of CC gradually tends to be younger. The mortality rate of young patients suffering from CC has a significant upward trend. Since there are no obvious abnormal symptoms in CC at early stage, patients often need to receive chemotherapy after surgery to more completely eliminate cancer cells. However, chemotherapy often brings certain adverse reactions, such as bone marrow suppression, vomiting, and nausea. With the extension of chemotherapy time, the patient's body tolerance decreases, which can easily lead to the occurrence of various adverse reactions.

Recently, the use of integrated traditional Chinese and Western medicine in patients has become a popular method for many scholars. TCM syndrome differentiation and evidence-based medicine helps improve the treatment effect and life quality of CC patients [30]. TCM has antivirus and strengthening the body's immunity effects and can avoid the trauma caused by physical and surgical treatment. Moreover, the prognosis of TCM is better. The systemic side effects of TCM are less [31]. Chemotherapy mainly uses poison to fight poison. Long-term treatment can cause fiery heat toxins to invade the patient's body. Therefore, qi and blood drugs should be used to improve the symptoms of such patients. In this study, *Astragalus*, *Atractylodes*, *Lycium barbarum*, and other traditional Chinese medicines can nourish the kidney and invigorate the spleen and kidney. Soil Fuling can clear away heat and detoxify. Psoralen can dispel blood stasis and masses.

In this study, the results showed that the clinical efficacy of traditional Chinese medicine combined with chemotherapy was better than chemotherapy alone. In addition, traditional Chinese medicine can improve the immune function and reduce the incidence of adverse reactions. Compared with chemotherapy alone, traditional Chinese medicine significantly reduced the serum tumor markers CA125, CEA, and TNF-*α* levels, immune index CD8+ levels, and adverse reactions. Traditional Chinese medicine also increased the degree of clinical symptom relief, immune index CD4+, CD4+/CD8+ levels, serum immunoglobulin IgG, IgA, and IgM levels, and life quality scores. The results show that traditional Chinese medicine can improve the tolerance and clinical efficacy of CC patients and reduce the toxic and side effects of chemotherapy. However, this study still has certain shortcomings. On the one hand, the research time is relatively short, and the patient's long-term tumor recurrence and quality of life are unknown. On the other hand, the number of selected cases is small, and the pathological type is single.

## 5. Conclusion

In summary, traditional Chinese medicine can help improve the clinical efficacy of chemotherapy and the serum tumor markers and immunoglobulin levels of CC patients. More importantly, this treatment method is feasible and safe, which is worthy of promotion.

## Figures and Tables

**Table 1:** Comparison of general clinical data between the two groups of patients (*n*).

Features	Observation (*n* = 39)	Control (*n* = 39)
Age	54.36 ± 10.03	52.91 ± 9.67
Course of disease (years)	2.6 ± 0.7	2.4 ± 0.8
The pathologic types		
Squamous cell carcinoma	30 (76.9%)	32 (82.0%)
Adenocarcinoma	6 (15.4%)	5 (12.8%)
Gland scale cancer	3 (7.7%)	2 (2.2%)
Stage		
II A	7 (18.0%)	6 (15.4%)
II B	11 (28.2%)	12 (30.8%)
III A	10 (25.6%)	10 (25.6%)
III B	11 (28.2%)	11 (28.2%)

**Table 2:** Comparison of short-term efficacy between the two groups of patients (*n* (%)).

Indicators	Observation	Control	*P*
CR	11 (28.2%)	9 (23.1%)	0.152
PR	24 (61.5%)	25 (64.1%)	
SD	3 (7.7%)	4 (10.2%)	
PD	1 (2.6%)	1 (2.6%)	
ORR	35/39 (89.7%)	34/39 (87.2%)	

**Table 3:** Comparison of immune function between the two groups.

Group	Time	CD4^＋^	CD8^＋^	CD4^＋^/CD8^＋^
Observation	Before treatment	27.35 ± 5.43	29.03 ± 6.10	0.99 ± 0.31
	After treatment	30.21 ± 5.03^*∗*#^	25.76 ± 4.71^*∗*#^	1.21 ± 0.30^*∗*#^

Control	Before treatment	27.78 ± 5.21	29.51 ± 6.32	0.98 ± 0.31
	After treatment	28.98 ± 4.86^*∗*^	27.63 ± 5.77	1.05 ± 0.29

Before and after treatment, ^*∗*^*P* < 0.05; compared with the control group, ^#^*P* < 0.05.

**Table 4:** Comparison of serum immunoglobulin levels between the two groups (g/L).

Group	Time	IgG	IgA	IgM
Control	Before treatment	11.03 ± 1.99	1.65 ± 1.16	1.39 ± 0.72
	After treatment	14.37 ± 2.17	2.07 ± 0.97	1.64 ± 0.73

Observation	Before treatment	11.23 ± 2.11	1.70 ± 1.21	1.42 ± 0.75
	After treatment	17.03 ± 2.74^*∗*^	2.67 ± 0.78^*∗*^	1.93 ± 0.83^*∗*^

Compared with the control group after treatment, ^*∗*^*P* < 0.05.

## Data Availability

The datasets used during the present study are available from the corresponding author upon request.
